# Peripheral brain-derived neurotrophic factor (BDNF) and salivary cortisol levels in college students with different levels of academic stress. Study protocol

**DOI:** 10.1371/journal.pone.0282007

**Published:** 2023-02-22

**Authors:** Juan-Luis Castillo-Navarrete, Alejandra Guzmán-Castillo, Claudio Bustos, Romina Rojas

**Affiliations:** 1 Departamento de Tecnología Médica, Facultad de Medicina, Universidad de Concepción, Concepción, Chile; 2 Programa de Neurociencia, Psiquiatría y Salud Mental, NEPSAM (http://nepsam.udec.cl), Universidad de Concepción, Concepción, Chile; 3 Programme in Mental Health, Facultad de Medicina, Universidad de Concepción, Concepción, Chile; 4 Departamento de Ciencias Básicas y Morfología, Facultad de Medicina, Universidad Católica de la Santísima Concepción, Concepción, Chile; 5 Departamento de Psicología, Facultad de Ciencias Sociales, Universidad de Concepción, Concepción, Chile; 6 Departamento de Farmacología, Facultad de Ciencias Biológicas, Universidad de Concepción, Concepción, Chile; Chiba Daigaku, JAPAN

## Abstract

**Introduction:**

Brain-derived neurotrophic factor (BDNF) is essential for brain physiological processes influencing memory and learning. BDNF levels can be affected by many factors, including stress. Stress increase serum and salivary cortisol levels. Academic stress is of the chronic type. BDNF levels can be measure from serum, plasma or platelets, and there is still no standard methodology, which is relevant to ensure reproducibility and comparability between studies.

**Hypothesis:**

(i) BDNF concentrations in serum show greater variability than in plasma. (ii) In college students with academic stress, peripheral BDNF decreases and salivary cortisol increases.

**General objective:**

To standardize plasma and serum collection for BDNF levels and to determine whether academic stress affects peripheral BDNF and salivary cortisol levels.

**Design:**

Quantitative research, with a non-experimental cross-sectional descriptive design.

**Participants:**

Student volunteers. Under convenience sampling, 20 individuals will be included for standardization of plasma and serum collection and between 70 and 80 individuals to determine the effect of academic stress on BDNF and salivary cortisol.

**Peripheral blood and salivary cortisol sampling, measurements:**

12 mL of peripheral blood (with and without anticoagulant) will be drawn per participant, separated from plasma or serum and cryopreserved at -80°C. Additionally, they will be instructed in the collection of 1 mL of saliva samples, which will be centrifuged. Val66Met polymorphism will be performed by allele-specific PCR, while BDNF and salivary cortisol levels will be determined by ELISA.

**Statistical analysis:**

(i) descriptive analysis of the variables, through measures of central tendency and dispersion, and the categorical variables through their frequency and percentage. (ii) Then a bivariate analysis will be performed comparing groups using each variable separately.

**Expected results:**

We expect to (i) determine the analytical factors that allow a better reproducibility in the measurement of peripheral BDNF, and (ii) the effect of academic stress on BDNF and salivary cortisol levels.

## Introduction

From a scientific viewpoint, replicability between different studies becomes an essential procedure. One criterion used to ensure replicability is the validity of knowledge [1, 2]. In this sense, achieving replicability between different studies is not a simple task. This is even more so when complex and multifactorial issues are addressed from different points of view. In this context, addressing the phenomenon of stress is a complex challenge, even more so in the college student population, who experience a particular type of stress, academic stress, many of whose scope is still unknown and the target of various investigations [3].

This protocol addresses the impact of academic stress in college students on peripheral levels of brain-derived neurotrophic factor (BDNF) and salivary cortisol levels. However, this is not an easy task, because on the one hand, although BDNF levels can be measured in serum, plasma, or platelets, there is no standard methodology, which is relevant to ensure reproducibility and comparability between studies [4–6]. Alternatively, the measurement of salivary cortisol, especially the variations in the conditions under which saliva sampling is performed, is not free from variability and consequently, discrepancies between different studies [7]. Consequently, to contextualize this protocol, information is provided below regarding (i) academic stress, (ii) BDNF and its assessment options in the periphery, and (iii) salivary cortisol.

### Academic stress

Stress is a neuroendocrine, immunological, and behavioral response of an organism to any imposed demand [8] (Selye, 1956). The triggering stressor of this adaptive response will produce an acute or chronic response [9, 10]. Acute stress produces increases in heart rate, blood pressure, respiratory rate, glycemia and coagulation factors [11]. This is followed by a coping stage and then a stage of relaxation, lowering activation levels, returning physiological homeostasis [10–12]. Chronic stress also has an alert phase, followed by a resistance stage. Finally, there is a phase of exhaustion due to the inability to cope with the stressor any longer, resulting in a homeostatic imbalance. This results in various pathophysiological phenomena, such as increased proinflammatory cytokines and acute phase proteins, structural changes in the hippocampus and various hormonal alterations [13–16]. Chronic stress has also been associated with the development and/or poor prognosis of highly prevalent pathologies such as cardiovascular disease and cancer [12, 17–20].

When a stressful experience originates in the context of an educational process, it is referred to as academic stress. It already occurs in students at the primary level and increases as the student progresses through the level of studies, reaching their highest grades at university [21, 22]. Higher education represents the peak of academic stress given the high workloads. But it also often coincides with the process of separating from the family, entering the labor market, and adapting to an unfamiliar environment [22, 23]. University students are subjected to activities with particularly stressful periods, which demand great adaptive efforts, causing them to experience exhaustion, little interest in studying and even difficulty in facing the challenges of the environment [24, 25].

Stress has been associated with the activation of the hypothalamic-pituitary-adrenal axis (HPA) which has been implicated in both mood regulation (particularly depression) and cognitive functioning. The HPA has stimulatory pathways as well as feedback loops that together regulate stress hormone production, including cortisol [12]. Additionally, not least, stress alters the sleep cycle, and partial or total loss of sleep alters the HPA response, producing increased cortisol [26, 27]. Exposure to mental or physical stress triggers the release of large amounts of cortisol to activate alert responses that increase blood glucose levels and modify mental reactions, thus being a biomarker directly correlated with stress levels [28–30]. In addition, stress has potent effects on BDNF [31, 32]. Thus, chronic stress stimulates the HPA by increasing the expression of cortisol, neuroinflammatory cytokines and decreasing BDNF levels [33]. Additionally, measuring peripheral BDNF levels in acute stress has been suggested to objectify the functioning of the HPA [27, 34, 35].

### BDNF and its assessment options in the periphery

BDNF is a protein that is widely distributed throughout the adult brain, in almost all cortical areas, as well as in several subcortical regions and regions of the spinal cord. It is essential for physiological functions such as modulation of dendritic branching, dendritic spine morphology, synaptic plasticity, and long-term potentiation (LTP), thus influencing memory, learning, appetite, and sleep [36–39]. The BDNF gene (located on 11p13) has a nonconservative exonic single-nucleotide polymorphism (SNP) at nucleotide 196 (dbSNP rs6265, G/A). This SNP changes valine (Val) to methionine (Met) within the proBDNF 5’ protein at codon 66 (Val66Met). This results in altered BDNF packaging in secretory granules, decreasing BDNF release-dependent activity. The Val/Met variant has been associated with cognitive impairments, impaired episodic memory, and decreased hippocampal activity [38, 40].

BDNF in peripheral blood (PB), derived from both platelets and the brain [37, 41, 42]. Because it can cross the blood-brain barrier, peripheral BDNF levels reflect brain BDNF levels and cortical integrity [41–43]. In PB, the determination of BDNF levels is performed in serum, plasma, or platelets [41, 44, 45].

Several publications have described significantly decreased BDNF levels in patients with major depression [46, 47], schizophrenia, bipolar disorders [48, 49] or autism spectrum disorders [50, 51]. To the aforementioned, we can add the increase in serum and plasma levels of BDNF in patients under treatment with antidepressants [47, 52–54] and in studies related to neurorehabilitation in schizophrenic patients [55] and in studies on the effect of aerobic exercise in patients with vascular accidents [33].

The quantification of BDNF can be performed by ELISA (enzyme-linked immunosorbent assay), and there are different options on the market; however, only recently have various methodological aspects been addressed in relation to the measurement of BDNF and proBDNF concentrations [41], especially about reproducibility, precision, and influence of the material from which the measurements are made and its manipulation (blood, tissue).

It is necessary to consider, that in addition to the known variables that can affect BDNF levels (drugs, smoking, body mass index), differences have been found in the way the sample to be analyzed was obtained, which adds a new variable to consider as a source of variation [41, 46, 56] and which is obviously an additional difficulty when assessing BDNF levels in relation to a given condition, pathology, or treatment [3]. The above has allowed explaining the discrepancies of reports with unequal results, in humans, depending on whether they determine BDNF levels in whole blood, serum or plasma [3, 41, 43, 57].

At present, there is a lack of a uniform methodology, let alone the existence of a consensus regarding the analytical considerations that allow BDNF levels to be compared [3]. It has been reported that many BDNF studies lack reproducibility, among other things, because of some factors that are not always considered, such as, for example, the pre-analytical treatment of the samples and reproducibility of the analytical method [4–6].

In this regard, during the pre-analytical phase, circadian variations, sample collection process, sample preparation and storage acquire great importance [41, 57]. In healthy adults, individual characteristics that have a specific impact on stored (platelet) and circulating (plasma) BDNF levels include parameters such as age, weight, sex, menstrual cycle, exercise, fasting, smoking alcohol consumption and living in an urban environment, as well as the timing of sampling [37, 56–62].

Adding to the above, and given that BDNF in plasma is generally detected in the pg/ml range, while serum is usually measured in the order of ng/ml [44, 45], a recurrent question arises among researchers: where to measure BDNF levels? In plasma? In the whole blood? In serum? In this regard, a tacit consensus has slowly emerged, supporting the choice of plasma as the most characteristic and stable source to measure BDNF levels, as well as the most representative of the total circulating BDNF [3, 41]. The above have not obeyed any agreement and have only been the consequence of several observations. First, the serum concentration of BDNF is approximately 100 times higher than plasma levels [41]. Second, serum BDNF concentration is affected by the blood sample handling because of the presence of platelets, which contain BDNF and their degranulatory processes, can secrete BDNF [41, 43]. During the blood collection, simple aspiration forces produced by the needle can cause platelet degranulation. Even changes in ambient temperature, as well as the time it takes the laboratory to separate the plasma, can cause BDNF release [41, 57]. Moreover, platelets have a half-life in circulation ranging from 8 to 12 days, whereas BDNF circulates in plasma for less than one hour [41]. Moreover, serum BDNF concentrations are almost identical to those obtained from washed platelet lysates [37, 41]. In turn, the determination of BDNF from whole blood, although the reported results are similar to those obtained in serum, involves a methodological step of cell lysis, which may add more variability [57].

The aforementioned factors, with the various pre-analytical variables related to peripheral blood sampling, handling, separation of components and their correct preservation, as well as analytical variables themselves (sample dilution, operator variations, commercial assays available, etc.), must necessarily be considered when examining the circulating levels of BDNF in various population samples with certain pathological and non-pathological characteristics [3]. Because of the above, an adequate standardization of reference or analytical methodological consensus becomes pertinent to ensure reproducibility and thus comparison between the various studies. Undoubtedly, the fact that the determination of BDNF in plasma has slowly positioned itself as the most representative and stable source for measuring BDNF levels, as well as the most representative of the total circulating BDNF, is an important step, but there are still many factors to consider (i) Constitutive: the presence of the Val66Met polymorphism, pathologies and addictions, age, sex, exercise, BMI, circadian variations and fasting. (ii) Pre-analytical: the standardization of sample collection either plasma or serum, with emphasis on the time spent before centrifugation, centrifugation time and speed, as well as sample preservation conditions. (iii) Analytical: the standardization of sample dilution and diluent used, the preparation of accessory reagents and the quality of commercially available assays (sensitivity and specificity) [3].

### Salivary cortisol

Cortisol is a hormone produced by the adrenal cortex by the activation of HPA. Its release follows a circadian rhythm, peaking at 30–60 min after awakening and then gradually tapering off over the day with its lowest levels at night [7, 63, 64]. In the presence of a stressor, the HPA axis is activated. This produces a hormonal chain response that subsequently releases cortisol into the bloodstream, with a peak in saliva approximately 25–30 min later [63]. Cortisol has widespread effects on physiological processes that prepare for and support coping with the stressor [63]. Numerous studies have related cortisol levels to the degree of stress and anxiety in adults and children subjected to different stressful situations [65–67]. The reference ranges of cortisol of healthy individuals in blood and saliva are 30–160 ng/mL and 1–1.6 ng/mL, respectively [68, 69].

Salivary cortisol has been widely used as a biochemical marker in stress research because saliva can be readily collected under different conditions and repeatedly throughout the day [7, 64, 70, 71]. The linear relationship between blood cortisol (free and bound to cortisol-binding globulin) concentrations and salivary cortisol concentrations is well established [7]. Obtaining saliva samples for cortisol determination is simple, non-invasive, and stress-free, whereas blood sampling may be stressful and thus elevate cortisol levels [70]. Much literature evidence indicates key biomarkers for the analysis of saliva samples, including cortisol, immunoglobulin A, lysozyme, melatonin, alpha-amylase, chromogranin A, FGF2 [66], and complementary ones such as 3-methoxy-4-hydroxyphenylglycol, testosterone, and its metabolite dehydroepiandrosterone [72]. These determinations in saliva have been preferred by several researchers because of the low invasiveness of sample collection and lower sampling costs. In this sense, salivary cortisol levels are independent of saliva flow rate and correlate with the biologically active, unbound fraction of plasma and serum cortisol [73]. Furthermore, the time lag between alterations of cortisol levels in plasma and saliva is 1 to 2 min [64, 70].

In acute stress situations, a sharp rise in cortisol has been identified, presenting a maximum 20–30 min after exposure to the stressor [68, 74], while a situation of sustained stress over time seems to trigger an overstimulation of the hypothalamic-pituitary-adrenal axis that causes its desensitization with a decrease in secreted cortisol levels, even below the values expected for healthy individuals [75, 76]. In turn, it is important to consider that cortisol concentration depends on sex, age, and population characteristics [77].

The cortisol awakening response (CAR), describes the marked increase in cortisol levels across the first 30–45 min following morning awakening [7, 64, 71, 74]. CAR is widely used to measure HPA dynamic function, and its associations with various pathologies, and physical and neurobiological variables have been studied extensively over the last two decades [64, 71]. Multiple strategies have been used to measure the cortisol response after awakening [64, 78]. However, the often used indices that capture the cortisol levels across serial measures are (i) area under the curve with respect to the base line, (ii) the area under the curve with respect to increase (AUCi), and (iii) the area under the curve with respect to ground (AUCg). Studies suggest that AUCi measures the change (i.e., increase or decrease) provoked by awakening, whereas AUCg reflects the total cortisol output after awakening [64, 71].

Studies investigating stress and cortisol secretion have generally been conducted under laboratory conditions, with the intention of being as representative as possible of ideal conditions. However, the difficulties of design and sampling are not minor, especially when considering studies under real-life conditions [7, 64]. It is in this sense that assessing individuals under natural conditions has been proposed as an affordable and interesting alternative, even for individuals under academic demands.

### Problem and research question

Based on the above, the research problem arises, given that although BDNF plays a key role in memory and learning, in addition to being directly related to the HPA, before considering its use as an indicator reflecting conditions (stress and/or neuropsychiatric pathology), it is necessary to better understand the variables that influence the preparation of the sample for its plasma and/or serum determination, given the influence that this has on the analytical phase itself [3, 41]. Furthermore, given that stress, on the one hand, affects the HPA, which is possible to evidence through alterations in cortisol levels, and on the other hand affects BDNF expression, it is possible to derive as research questions: (i) Are there differences in the variability of peripheral BDNF measurement depending on the procedure for obtaining plasma and serum? and (ii) Does academic stress produce alterations in salivary cortisol levels and in peripheral BDNF levels?

While it is possible to assume, based on the available literature, that academic stress will lead to a decrease in peripheral BDNF levels, this may not necessarily be the case. In this regard, it has been reported that serum BDNF levels in schizophrenics are significantly decreased compared to healthy individuals and that when faced with cognitive training, these serum levels increased significantly, which would indicate that processes related to the specific demands of the cognitive training condition induce an increase in serum BDNF levels, but not an increase in cognitive ability [55]. Consequently, these authors suggest that serum BDNF levels could serve as a peripheral biomarker for the specific effects of cognitive training [55]. In line with this, in our experience we have found changes in plasma BDNF levels in college students, which can be either increasing or decreasing, depending on the individual, especially considering that college studies involve a high cognitive activity [3].

In this sense, this project becomes an opportunity to obtain scientific information that allows an approach to the standardization of the processing of both serum and plasma samples for the determination of BDNF in peripheral blood. In addition to objectifying the effects of academic stress on salivary cortisol levels and peripheral BDNF levels in the university student population. This information is relevant to consider not only in institutional educational models and student support programs, but also in the understanding of the phenomenon of academic stress from an integral view of the network of regulatory mechanisms formed by the psychological, neurological, immunological and endocrinological systems.

## Method

Considering that the research question is the existence of a large difference in the variability of peripheral BDNF measurement depending on the procedure for obtaining plasma and serum and that academic stress would produce alterations in salivary cortisol levels and in peripheral BDNF levels, it is pertinent to propose as research hypotheses: (i) The BDNF concentrations obtained in serum samples show greater variability than those obtained in plasma samples in college students. (ii) In college students under academic stress, there is a decrease in plasma and serum BDNF levels and an increase in salivary cortisol levels.

### General aim

To standardize the procedure for obtaining plasma and serum for BDNF determination in college students and to determine whether academic stress affects peripheral BDNF and salivary cortisol levels.

### Specific aims

(i) To determine BDNF levels in peripheral blood under different plasma and serum collection conditions. (ii) To determine peripheral BDNF levels in college students with different levels of academic stress. (iii) To determine salivary cortisol levels in college students with different levels of academic stress.

### Methodology

A quantitative methodology is proposed given that data are obtained on the basis of peripheral blood and saliva sampling and the application of questionnaires to the participating subjects.

### Study design

Quantitative research with a transversal non-experimental design is proposed. The research is non-experimental since it will be conducted out without deliberately manipulating the variables, observing the phenomena as they occur in their natural context, and then analyzing them.

### Universe and sample

The universe will be given by the totality of undergraduate students of the Faculty of Medicine of the Universidad de Concepción (UdeC) and the Universidad Católica de la Santísima Concepción (UCSC). A convenience sample will be obtained, based on the literature (Girard et al., 2020) and the availability of resources, estimated a number between 70 and 80 individuals.

### Procedure

#### Participants

Correspond to students of the Faculty of Medicine of the UdeC and the UCSC (excluding first year students), who will participate voluntarily by signing an informed consent form. In the case of specific objective 1 (To determine BDNF levels in peripheral blood under different conditions of sample collection), under a convenience sampling, 20 individuals will be included and for specific objectives 2 and 3 (To determine peripheral BDNF and salivary cortisol levels in college students with different levels of academic stress) also under a convenience sampling, a number of between 70 and 80 individuals (50% women and 50% men) will be included.

#### Timing

Recruitment and sample collection will occur at least two months into the corresponding academic semester. In this way, it is expected that the phenomenon of academic stress will be present (Castillo 2020).

#### Recruitment

Students will be invited to participate in this study using a mass email sent by the degree program heads, and those who agree to participate will be asked for their contact details in order to coordinate the taking of peripheral blood samples and the delivery of saliva collection tubes (with prior training).

#### Inclusion criteria

For recruitment and sample collection, students from UdeC and UCSC will be included if they freely express their willingness to participate by signing an informed consent form, responding to psychometric instruments, filling out a general data form and having a peripheral blood and salivary cortisol sample taken correctly.

#### Exclusion criteria

Considering, on the one hand, the premise of evaluating college students under conditions of academic stress, in natural (real) conditions and, on the other hand, the different variables that mainly affect cortisol levels, individuals who report (i) being affected by any psychiatric disorder will be excluded from the analysis, (ii) any chronic illness, (iii) undergoing psychological, (iv) pharmacological treatment, (v) smoking, (vi) drinking alcohol, (vii) being physically active regularly, (viii) having done so during the last 48 h, (ix) sleeping less than 7 h the night before the sample was taken, and in the case of women (x) taking oral contraceptives.

#### Instruments for data collection

(i) The Self-Report Symptom Questionnaire (SRQ 20), a non-specific scale on somatic complaints and global level of functioning, will be applied, evaluating anxious and depressive symptoms, with a maximum possible score of 20 points (Annex 1 in S1 File). This questionnaire only discriminates between two categories: present and absent. In Chile it has been validated with cut-off points of 8 points and above, values with which the individual can be considered as a probable case of high risk of suffering from a mental disorder [3, 79, 80]. (ii) For the evaluation of academic stress, the SISCO-II academic stress inventory (Annex 2 in S1 File) will be applied, which shows very good psychometric properties in our country [3, 24, 25, 81]. (iii) In addition, participants will be asked to complete a general data form (Annex 3 in S1 File), which aims to identify those who suffer from a psychiatric disorder and/or are undergoing psychological treatment, in addition to obtaining information about the consumption of medication, tobacco, alcohol and physical activity, relevant information at the time of analyzing and interpreting the results derived from this research.

#### Peripheral blood sample extraction

To fulfill specific objective 1 (to determine BDNF levels in peripheral blood under different sample collection conditions), 12 ml of peripheral blood (04 tubes) will be extracted from each participant using 02 tubes with EDTA K3 as anticoagulant and 02 tubes without additives (to obtain serum). To fulfill the specific objective 2 (To determine peripheral BDNF levels in college students with different levels of academic stress) each participant will have 6 mL of peripheral blood (02 tubes) obtained from clean puncture using 01 tubes with EDTA K3 as anticoagulant and 01 tube without any additive. The phlebotomy process will be performed by a trained professional on the premises of the Medical Technology Teaching Laboratories, UdeC and UCSC.

#### Obtaining serum and plasma (Fig 1)

To fulfill specific objective 1 (to determine BDNF levels in peripheral blood under different sample collection conditions) the peripheral blood samples obtained will be centrifuged for 10 min at 2500 g and then aliquoted, either plasma or serum, and stored at -80°C until processing. In the case of samples with EDTA K3 as the anticoagulant, one tube will be centrifuged as soon as the blood sample is extracted and the other tube after 30 min at room temperature and in the case of samples without anticoagulant, one tube will be centrifuged after 10 min at room temperature and the other after 30 min. For fulfilling specific objective 2 (to determine approximate serum and plasma reference values of BDNF in college students) the samples will be centrifuged according to the results of specific objective 1, and then aliquoted and stored at -80°C until processing. These procedures will be carried out at the Medical Technology Teaching Laboratories, UdeC and UCSC.

**Fig 1:**
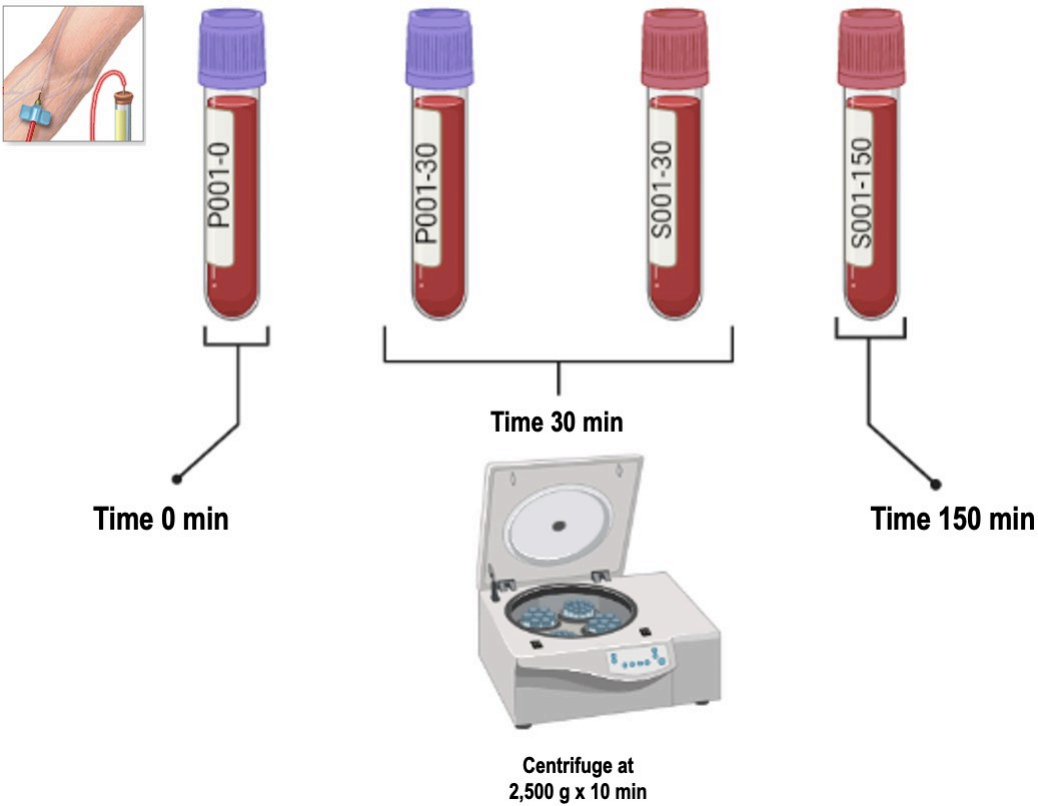
Schematic of both the labeling procedure and the centrifugation of each tube in which the peripheral blood sample will be obtained. Each participant will have 12 ml of peripheral blood (04 tubes) drawn using 02 tubes with EDTA K3 as anticoagulant and 02 tubes without additives (to obtain serum). The peripheral blood samples obtained will be centrifuged for 10 min at 2500 g and then either plasma or serum will be aliquoted and stored at -80°C until processing. In the case of samples with EDTA K3 as the anticoagulant, one tube shall be centrifuged as soon as the blood sample is withdrawn and the other tube after 30 min at room temperature. For samples without anticoagulant, one tube shall be centrifuged after 10 min at room temperature and the other after 30 min.

#### Obtaining a salivary sample

Each participant will be instructed how to collect saliva samples (approximately 1mL) in sterile 15 mL propylene tubes (Salivettes®) [82], which will be immediately refrigerated. This ensures stable maintenance of cortisol in saliva for one week [83, 84]. They are then stored in the laboratory at -20°C until analysis. For proper sampling, individuals should not ingest food, chew gum, or brush their teeth, for a period of at least 60 min before saliva sample collection. Due to the fluctuation of cortisol levels during the day, samples will be taken at three different times 08:00 AM, 14:00 PM and 20:00 PM.

#### Serum and plasma determination of BDNF and salivary cortisol

Based on previous experience [3], with stored plasma and/or serum, BDNF levels will be determined by ELISA using the commercial assay "Human BDNF PicoKine™ ELISA Kit" (EK0307) solid-phase immunoassay specially designed to measure human BDNF [85]. Final absorbances will be measured on the Infinite® 200 PRO NanoQuant instrument at a wavelength of 450 nm. All determination will be performed in triplicate for specific target 1 and in duplicate for specific target 2 and concentrations will be reported in pg/ml. For salivary cortisol, Saliva samples will be thawed and heated at 90°C to inactivate contaminating viruses such as Sars-Cov-2 [86]. They are then centrifuged at 10,000 xg for 4 min. Detection is performed on the supernatant by ELISA using the commercial assay Cortisol Saliva ELISA Assay Kit (Eagle Bioscience, CRT32-K01) [87] and microplate reader detection at 405 nm, with a correction between 570 and 590 nm [88]. For the analysis we will consider the AUCi, and the AUCg [64, 71]. All these determinations will be performed at the Molecular Psychiatry and Genetics Laboratory, belonging to the Department of Psychiatry and Mental Health of the Faculty of Medicine of the Universidad de Concepción, attached to the Neuroscience, Psychiatry and Mental Health Program (NEPSAM UdeC).

#### Determination of Val66Met polymorphism

DNA extraction from peripheral blood will be performed using the commercial assay "Blood DNA Preparation Kit" (Jena Bioscience, PP205S) which is based on a cell lysis step to subsequently precipitate proteins, with subsequent hydration of the DNA obtained [89]. Once the DNA is extracted, it will be stored at -80°C until use. The determination of the Val66Met polymorphism of the BDNF gene will be studied by allele-specific PCR, for which specific primers will be designed to identify by conventional PCR each different type of alleles present (homozygous or heterozygous) [90].

#### Data analysis proposal

(i) In the first instance, a descriptive analysis of the variables will be carried out, through measures of central tendency and dispersion, and the categorical variables through their frequency and percentage. (ii) Then a bivariate analysis will be carried out considering the comparison of groups using each variable separately, the chi-square test will be used if the variable under study is categorical and the Student’s t-test (Mann-Whitney, if the distributional assumption of normality is not met) if the variable under study is numerical. Pearson’s product-moment correlation coefficient will be used to determine the relationship between numerical variables. In this project, we will work with a significance level of α = 0.05. The Excel 2016 software will be used to generate the databases. The R program [91] will be used for the statistical analysis.

#### Ethical considerations

The authors assert that all procedures contributing to this work will comply with the ethical standards of the relevant national and institutional committees on human experimentation and with the Helsinki Declaration of 1975, as revised in 2008, allowing test subjects to voluntarily participate in the research and to abandon it when they wish, without having to provide any justification. All procedures involving human subjects/patients were approved by the Ethics, Bioethics and Biosafety Committee of the Vice-Rectory of Research and Development of the Universidad de Concepción (CEBB 1172–2022). Each person who agrees to participate will sign an informed consent form and will be completely anonymized and cannot be identified.

## Discussion

Considering stress as a complex phenomenon given the participation of the different organ systems that make up an individual and, consequently, its wide spectrum of psychosomatic manifestations, academic stress in college students can be considered a phenomenon that makes those who suffer from it particularly susceptible to the development of mental disorders (e.g. anxiety disorders and depression), given that they are frequently subjected to a homogeneous activity with particularly stressful periods, more so than at other educational levels. Moreover, academic stress is a reality at the student level that is not always objectified and difficult to measure, affecting not only academic performance, e.g., through its consequences on memory and learning, but also the quality indicators of the educational institution.

Furthermore, stress can have potent effects on neurotrophin expression, in particular on BDNF and that these effects are highly sensitive to the form, duration and timing of stress, as well as to the sex of the subject receiving the stress [31]. From the occupational viewpoint, it has been reported that serum levels with occupational stress interact to produce occupational burnout and precisely those who experience this burnout are those with lower serum levels of BDNF [32]. Furthermore, it has been suggested that elevated BDNF levels would have a buffering effect on job burnout when stress levels are still at low levels; however, when this increase, the protective effects of BDNF would be lost [32].

The adrenal sympathetic system along with the HPA is activated by psychological stress by increasing the expression of cortisol and neuroinflammatory cytokines and decreasing BDNF levels [92]. It has been suggested to measure peripheral BDNF levels during acute stress to objectify the functioning of the HPA given the reciprocal interaction of BDNF and cortisol pathways, i.e., when decreases in peripheral BDNF levels occur, there are increases in cortisol levels [34]. It should be added, that chronic stress leads to a dysregulation of the hypothalamic-pituitary-adrenal axis, producing decreased BDNF levels [27, 35, 93], which has been described both in animal studies [27, 94] and in several studies related to stress-associated mood disorders [27, 36, 95].

To date, there are no studies describing what happens to BDNF levels in academic stress, which could be important for the understanding of this phenomenon, especially if we consider the processes in which BDNF participate, fundamentally memory, cognition, and learning, especially when considering that peripheral BDNF levels reflect brain BDNF levels and are positively correlated [36, 42, 96].

However, there are discrepancies in reports, with unequal results, depending on whether BDNF levels are determined in whole blood, serum, or plasma [3, 41, 43, 57].

From the sample viewpoint, in the periphery, BDNF can be found circulating in plasma and platelets. During the blood coagulation process, platelets are activated and release their BDNF content, so serum BDNF concentrations reflect both plasma BDNF and BDNF produced by platelet degranulation, which is why plasma BDNF concentrations (measured in pg/L) are lower than serum BDNF concentrations (measured in ng/mL) [3–5, 97]. In the case of plasma BDNF concentrations, these can be increased up to sevenfold depending on the delay in centrifugation of samples following sample collection [5, 6]. Although EDTA prevents coagulation, it has been shown that it does not completely stop platelet degranulation, implying that as sample centrifugation is delayed, there is an increase in plasma BDNF concentrations [5, 57]. Alternatively, serum BDNF concentrations increase during the first 30 min after blood collection, while the clotting process occurs to apparently remain stable after that [5, 6, 61]. Highly variable factors among different studies (and not always reported) refer to the conditions of coagulation duration and temperature, in addition to the duration and speed of sample centrifugation [5, 61]. The above is directly due to platelet BDNF and its consequent release due to the platelet degranulation process, as well as the physical stress they are subjected to (temperature and centrifugation) [61]. However, platelet degranulation (but not their aggregation) would be the critical step in this process since BDNF released by platelets would be possibly found in serum obtained either at 37°C for 10 min or at room temperature for 30 min or in turn, BDNF levels in serum that would reflect its release during platelet aggregation, would be obtained with a longer coagulation time, greater than one hour at 37°C or greater than two hours at room temperature [98].

A not minor point of the analytical phase in the determination of BDNF is how this will be performed. It has been reported that a fundamental factor to consider is the choice of the commercial assay to be used since depending on the manufacturer, there will be differences in the way they react to pro-BDNF and mature BDNF proteins, which is necessary for consideration when evaluating the ability to detect and accurately differentiate both forms [3, 97, 98], in addition to there being variations in the execution of the assay, either in reagent preparation or operator-dependent variations [4, 41]. Depending on the commercial assay, measurements can be influenced by the sample used, either plasma or serum, and there are not international recommendations or standard operating procedures regarding this [3, 99]. Polacchini et al. reported that 4 of 5 commercial ELISA assays studied had low-inter-assay reproducibility and only one commercial assay showed low-intra-assay coefficient of variation [41].

From the above, an analytically important point arises and was recently demonstrated [41]: while most available commercial ELISA assays can detect BDNF within the range of concentrations found in a healthy Caucasian population, this is not always possible, especially when evaluating patients. It is for this reason, manufacturers recommend working with diluted serum samples, there being no single value for this, so recommendations fluctuate between a 1:2 dilution up to 1:200 dilutions or higher if necessary [3, 41].

Another factor that can become a critical issue when considering specific clinical applications of serum BDNF determination is the ability of commercially available assays to distinguish between proBDNF and mature BDNF [3, 41]. Therefore, when considering studies that relate either mature BDNF and/or proBDNF, caution should be exercised in their interpretation due to the limited specificity of the antibodies used in the determination of the latter, which may correspond to as little as one-third of the concentration of mature BDNF [3, 100, 101].

Considering all the above, this project becomes an opportunity to obtain scientific information that allows an approach to the standardization of the processing of both serum and plasma samples for the determination of BDNF in peripheral blood. In addition to objectifying the effects of academic stress on salivary cortisol levels and peripheral BDNF levels in the college students population. This information is relevant to consider not only in institutional educational models and student support programs, but also in the understanding of the phenomenon of academic stress from an integral view of the network of regulatory mechanisms formed by the psychological, neurological, immunological and endocrinological systems.

Consequently, it is expected on the one hand to achieve a standardization of the procedure for obtaining serum and plasma for measuring BDNF in peripheral blood and on the other hand to establish the levels of peripheral BDNF and salivary cortisol in college students with different levels of academic stress. This is necessary and of great importance to continue with studies aimed at understanding the phenomenon of academic stress and to answer issues related to the effect that various conditions related to the mental health of college students (academic stress, adaptive disorders, addictions, etc.) would exert on peripheral BDNF levels and salivary cortisol levels. We intend to apply to Chilean funding sources external to UdeC, such as (i) Fondo de Investigación y Desarrollo en Salud (FONIS), (ii) Fondef IdeA (ANID) (iii) Fondecyt (ANID) or (iv) an eventual international competitive fund.

As an important part of the process of dissemination of results, an approach to both the Directorate of Student Affairs (DISE) of the UdeC and the Directorate of Student Services (DAE) of the UCSC is planned, with the aim of contributing to the process of mental health literacy that allows students to know what academic stress is, its immediate and long-term consequences on the health of those who suffer from it and the importance of incorporating attitudes and self-care measures for its prevention. In addition, the student centres will be involved in the dissemination of the aforementioned material so that they can share it on their different social network accounts. Additionally, to reach out to the community outside the universities, it is proposed to create digital information posters aimed at secondary schools in the city, which will focus on prevention and the harmful effects of academic stress.

Undoubtedly, this project has several limitations, including the aforementioned variations in sampling conditions for both BDNF and salivary cortisol (and the conditions of the individuals sampled). To this, we can add that apart from resource limitations, there is a small number of individuals to be studied. This will necessarily have to be replicated with new studies on a larger number of individuals. Another relevant aspect is that the determinations of both salivary cortisol and peripheral BDNF will be carried out, in each case, with a single commercial supplier, which implies that the replicative studies will have to be carried out under the same conditions. The variability of the different commercial assays for peripheral BDNF determination has already been discussed, and this will be a future challenge, especially once international collaborative contacts are established. Finally, from a population viewpoint, this study will only focus on one city in Chile, so it would be expected to find differences in stress levels in college students from other countries.

## Supporting information

S1 File (ZIP)

S2 File (PDF)
