# Supplementary material for: Peripheral brain-derived neurotrophic factor (BDNF) and salivary cortisol levels in college students with different levels of academic stress. Study protocol
Source: PLoS One. 2023 Feb 22;18(2):e0282007. doi: 10.1371/journal.pone.0282007 (PMC9946253; doi:10.1371/journal.pone.0282007)
Supplement: S1 File — (ZIP) [file pone.0282007.s001.zip › Annex/Annex 2.pdf]

GENERAL DATA

Gender ☐ Female ☐ Male Email ________________________________

Date of Bird (dd/mm/yyyy) _______/_______/_______

Career ____

University __________________________________ Semester ___________________________

SISCO-II INVENTORY OF ACADEMIC STRESS

The main objective of this questionnaire is to identify the characteristics of the stress that usually accompanies middle, high school, college and graduate students during their studies. The sincerity with which you answer the questions will be essential for the research. The information you provide will be completely confidential and only global results will be handled. The questionnaire is voluntary and you have the right to answer it or not.

1. During this semester, have you had moments of worry or nervousness?

   ☐ Yes

   ☐ No

2. With the idea of obtaining greater precision and using a scale from 1 to 5, indicate your level of worry or nervousness, where (1) is a little and (5) a lot.

1	2	3	4	5

On a scale from (1) to (5), where (1) is never, (2) is rarely, (3) is sometimes, (4) is almost always, and (5) is always, indicate how often the following situations bothered you:

Stressors					
	(1)	(2)	(3)	(4)	(5)
	Never	Rarely	Some of the time	Almost Always	Always
3.1 Overload of homework and academic assignments					
3.2 The personality and character of professors					
3.3 Teachers' evaluations (exams, essays, research papers, etc.)					
3.4 The type of work that professors ask you to do (consulting maps, worksheets, essays, concept maps, etc.)					
3.5 Not understanding the topics covered in class.					
3.6 Class participation (answering questions, presentations, etc.).					
3.7 Limited time to work					
3.8 Group mates make faster progress on assignments and/or academic work					

4. On a scale from (1) to (5), where (1) is never, (2) is rarely, (3) is sometimes, (4) is almost always, and (5) is always, point out how often you had physical, psychological, and behavioral reactions when you were worried or nervous.

Total Reaction: Physical and Psychological Reactions					
	(1)	(2)	(3)	(4)	(5)
	Never	Rarely	Some of the time	Almost Always	Always
4.1 Sleep disturbances (insomnia or nightmares)					
4.2 Chronic fatigue (permanent tiredness)					
4.3 Headaches or migraines					
4.4 Digestion problems, abdominal pain or diarrhea					
4.5 Scratching, nail-biting, rubbing, etc.					
4.6 Drowsiness or increased need for sleep.					
4.7 Muscle aches and/or contractures.					
4.8 Skin reactions (rash, peeling, etc.).					
4.9 Restlessness (inability to relax and be calm)					
4.10 Anxiety, anguish or desperation					
4.11 Increased or decreased food intake					
**Total Reaction: Social Behavioral Reactions**					
4.12 Feelings of depression and sadness (downcast)					
4.13 Feelings of aggressiveness or increased irritability					
4.14 Sudden mood swings					
4.15 Conflicts or tendency to argue or quarrel					
4.16 Isolation from others					
4.17 Unwillingness to perform your work as a student					

5. On a scale from (1) to (5), where (1) is never, (2) is rarely, (3) is sometimes, (4) is almost always, and (5) is always, indicate how often you used the following strategies to cope with the situation that caused you worried nervous.

	(1)	(2)	(3)	(4)	(5)
	Never	Rarely	Some of the time	Almost Always	Always
5.1 Making a plan for executing your tasks.					
5.2 Praising yourself					
5.3 Venting and confiding (verbalizing the situation that was causing you concern)					
5.4 Attempted to take something positive or beneficial out of a stressful situation					
5.5 Practicing a hobby (physical activity, reading, watching series, social networking, etc.)					
5.6 Accompanied by a loved one (family, pets, friends, etc.)					
