# Supplementary material for: Peripheral brain-derived neurotrophic factor (BDNF) and salivary cortisol levels in college students with different levels of academic stress. Study protocol
Source: PLoS One. 2023 Feb 22;18(2):e0282007. doi: 10.1371/journal.pone.0282007 (PMC9946253; doi:10.1371/journal.pone.0282007)
Supplement: S1 File — (ZIP) [file pone.0282007.s001.zip › Annex/Annex 3.pdf]

GENERAL DATA

Do you suffer from any psychiatric disorder (depression, bipolar, etc.)?	☐ Yes	☐ NO
Are you currently undergoing psychological treatment?	☐ Yes	☐ NO
Are you currently being treated with any medication?	☐ Yes: Indique cual(es) ______________________ ______________________	☐ NO
Do you smoke regularly?	☐ Yes: Indique N° de cigarrillos por semana ___________	☐ NO
Do you consume alcohol?	☐ Yes: Indicate Nº cigarettes per week_____________	☐ NO
Do you regularly engage in physical activity? Which one? ___________________________	☐ Yes: Indicate N° of times per week _____________	☐ NO

If you are a woman, please answer:

Do you use oral contraceptives?	☐ Yes	☐ NO
Indicate the date of your last menstrual period	____/_____/_____ (dd/mm/yyyy)	
