# Supplementary material for: Peripheral brain-derived neurotrophic factor (BDNF) and salivary cortisol levels in college students with different levels of academic stress. Study protocol
Source: PLoS One. 2023 Feb 22;18(2):e0282007. doi: 10.1371/journal.pone.0282007 (PMC9946253; doi:10.1371/journal.pone.0282007)
Supplement: S1 File — (ZIP) [file pone.0282007.s001.zip › Annex/Annex 1.pdf]

Self-Reporting Symptom Questionnaire (SRQ 20)

Below you will find a list of complaints that you may or may not have felt. If you have felt the discomfort described, mark an "X" in the box corresponding to "Yes", otherwise mark an "X" in the box corresponding to "NO".

**Symptoms**	**Yes**	**No**
**1.** Do you have frequent headaches?		
**2.** Do you have a poor appetite?		
**3.** Do you sleep poorly?		
**4.** Are you easily frightened?		
**5.** Do you suffer from hand tremors?		
**6.** Do you feel nervous, tense or bored? 7.		
**7.** Do you suffer from poor digestion?		
**8.** Do you have trouble thinking clearly?		
**9.** Do you feel sad?		
**10.** Do you cry very often?		
**11.** Do you have difficulty enjoying your daily activities?		
**12.** Do you have difficulty making decisions?		
**13.** Do you have difficulty doing your daily work (do you suffer with your work?)?		
**14.** Are you unable to play a useful role in your life?		
**15.** Have you lost interest in things?		
**16.** Do you feel that you are a useless person?		
**17.** Have you ever had the idea of ending your life?		
**18.** Do you feel tired all the time?		
**19.** Do you have unpleasant feelings in your stomach?		
**20.** Do you get tired easily?		
