# Supplementary material for: Peripheral brain-derived neurotrophic factor (BDNF) and salivary cortisol levels in college students with different levels of academic stress. Study protocol
Source: PLoS One. 2023 Feb 22;18(2):e0282007. doi: 10.1371/journal.pone.0282007 (PMC9946253; doi:10.1371/journal.pone.0282007)
Supplement: S2 File — (PDF) [file pone.0282007.s002.pdf]

Plan overview Data management*

Administrative Data	
Project name	Peripheral brain-derived neurotrophic factor (BDNF) and salivary cortisol levels in college students with different levels of academic stress. Study protocol.
ID	
Funding agency and Grant number	This work will be supported by Grant: 2021000311MUL of the Vice Rector of Research and Development (VRID) of the Universidad de Concepción; DIREG 01/2021 Directorate of Research of the Universidad Católica de la Santísima Concepción.
Language	English and Spanish
Principal Investigator (PI), identifier and contact details	• Castillo-Navarrete, Juan-Luis; ORCID iD: https://orcid.org/0000-0002-7401-2384; Associate Professor, Departamento de Tecnología Médica, Facultad de Medicina, Universidad de Concepción; Phone: 56998463346, Email: jucastillo@udec.cl ; Programa de Neurociencia, Psiquiatría y Salud Mental, NEPSAM (http://nepsam.udec.cl), Universidad de Concepción.<br>• Guzmán-Castillo, Alejandra; ORCID iD: https://orcid.org/0000-0002-0819-4650; Assistant Professor, Departamento de Ciencias Básicas y Morfología, Facultad de Medicina, Universidad Católica de la Santísima Concepción; Phone: 56982397954, Email: aleguzman@ucsc.cl ; Programa de Neurociencia, Psiquiatría y Salud Mental, NEPSAM (http://nepsam.udec.cl), Universidad de Concepción.
Co-investigators (with identifier)	• Bustos N., Claudio; ORCID iD: https://orcid.org/0000-0003-3478-9858. Email: clbustos@udec.cl<br>• Rojas P., Romina; ORCID iD: https://orcid.org/0000-0001-8209-6453, Email: romrojas@udec.cl
Project duration	24 months
Project description	Brain-derived neurotrophic factor (BDNF) is essential for brain physiological processes influencing memory and learning. BDNF levels can be affected by many factors, including stress. Stress increase serum and salivary cortisol levels. Academic stress is of the chronic type. BDNF levels can be meassure from serum, plasma or platelets, and there is still no standard methodology, which is relevant to ensure reproducibility and comparability between studies.<br>Hypothesis: (i) BDNF concentrations in serum show greater variability than in plasma. (ii) In college students with academic stress, peripheral BDNF decreases and salivary cortisol increases.<br>General objective: To standardize plasma and serum collection for BDNF levels and to determine whether academic stress affects peripheral BDNF and salivary cortisol levels.<br>Design: quantitative research, with a non-experimental cross-sectional descriptive design.<br>Participants: Student volunteers. Under convenience sampling, 20 individuals will be included for standardization of plasma and serum collection and between 70 and 80 individuals to determine the effect of academic stress on BDNF and salivary cortisol.<br>Peripheral blood and salivary cortisol sampling, measurements: 12 mL of peripheral blood (with and without anticoagulant) will be drawn per participant, separated from plasma or serum and cryopreserved at -80ºC. Additionally, they will be instructed in the collection of 1 mL of saliva samples,

	which will be centrifuged. Val66Met polymorphism will be performed by allele-specific PCR, while BDNF and salivary cortisol levels will be determined by ELISA.<br>Statistical analysis: (i) descriptive analysis of the variables, through measures of central tendency and dispersion, and the categorical variables through their frequency and percentage. (ii) Then a bivariate analysis will be performed comparing groups using each variable separately.<br>Expected results: We expect to (i) determine the analytical factors that allow a better reproducibility in the measurement of peripheral BDNF, and (ii) the effect of academic stress on BDNF and salivary cortisol levels.<br><br>Keywords: BDNF, plasma BDNF, serum BDNF, salivary cortisol, academic stress.
Date of first version	September 15, 2022
Date of latest version	February 03, 2023

**Data collection**	
What data will you collect or create?	• Information will be collected directly from the participants, so through the application of psychometric instruments, information will be obtained on levels of academic stress (stressors, symptomatology and coping) and depressive anxiety symptomatology, as well as general socio-demographic data.<br>• In addition, information about peripheral BDNF levels and the presence of the Val66Met polymorphism will be obtained from peripheral blood samples.<br>• Finally, information about saliva cortisol levels will be obtained from saliva samples.
How will the data be collected or created?	• Levels of academic stress will be obtained from the application of the SISCO-II inventory.<br>• Information on anxious-depressive symptomatology will be obtained from the application of the SRQ20 inventory.<br>• Sociodemographic information will be obtained by means of a question sheet on general data.<br>• The presence of the Val66Met polymorphism will be obtained by allele-specific PCR.<br>• Peripheral BDNF levels will be obtained by ELISA (duplicate determinations).<br>• Salivary cortisol levels will be obtained by ELISA (duplicate determinations).
Frequency of new data collection	As this is a cross-sectional study, information will be obtained from the participants once at the beginning of the project (application of surveys and collection of biological samples). However, during the development of the project, the corresponding laboratory determinations will be carried out.
Quantity of data to be generated	800 to 900 MB of data.
Data formats	Excel files (xlsx), Word files (docs), images (jpg, png, tif), Posit files (R-studio), PDF files.
What documentation and metadata will accompany the data?	Instruction file describing the different types of data and their corresponding coding (codebook).

Ethics and Legal Compliance	
How will you manage the ethical aspects of your research?	• All procedures with human subjects/patients were approved by the Ethics, Bioethics and Biosafety Committee of the Vice-Rectory of Research and Development of the University of Concepción (CEBB 1172-2022).<br>• Each person who agrees to participate will sign an informed consent form and will be completely anonymized and cannot be identified.<br>• The information collected will be kept confidential and a code will be used to identify the information provided by the research subjects, thus generating an anonymous database.
How will you manage intellectual property rights and copyright issues?	• Only the research team and the Ethics, Bioethics and Biosafety Committee of the Vice-Rectory of Research and Development of the Universidad de Concepción will have access to the data related to the study.<br>• The responsible researchers will be in charge of the custody of all the information of the study.<br>• The data generated will be the property of the Universidad de Concepción and the Universidad Católica de la Santísima Concepción.<br>• The Vicerrectoría de Investigación y Desarrollo de la Universidad de Concepción and the Dirección de Investigación de la Universidad Católica de la Santísima Concepción and the project reference (Grant 2021000311MUL and DIREG 01/2021, respectively) will be referenced in all works derived from the development of the project.<br>• Patentable results will be protected.<br>• When results are published in scientific journals, they will be subject to licensing or editorial policy restrictions.

Storage and Backup	
How will the data be stored and backed up during the research?	• The instrument answer sheets will be stored in folio folders in a locked cabinet in the office of the responsible investigator(s).<br>• Peripheral blood samples will be stored at -80°C and -20°C, respectively.<br>• The different files (spreadsheets, Excel, Word documents, images, Posit files, PDF) will be contained in folders and subfolders, which will be named according to their content.<br>• The data will be stored backed up:<br>○ in the cloud system Dropbox (password protected)<br>○ In the cloud system iCloud (password protected)<br>○ On external hard disk owned by each responsible researcher.
How will you manage access and security?	Access to cloud data will depend on authorisation and password sharing, which will only be available to authorised persons for research purposes only.

Selection and Preservation

Which data are of long-term value and should be retained, shared, and/or preserved?	• All research data will be stored for at least 10 years in the cloud (Dropbox and iCloud) and on external hard disk owned by each responsible researcher.<br>• Serum and plasma samples will be stored for at least 10 years at -80ºC.<br>• Saliva samples will be stored for two years at -20ºC.
What is the long-term preservation plan for the dataset?	All research data, together with serum and plasma samples, shall be stored for at least 10 years, while saliva samples shall be stored for 2 years.

Data Sharing	
How will you share the data?	• All data resulting from the development of this project will be available in scientific communications presented at conferences and in manuscripts to be published in peer-reviewed scientific journals.<br>• As far as possible, they will be open access or, once the data have been submitted, they will be made available to the public through the institutional repository of the University of Concepción (https://datav.udec.cl/).<br>• In addition, they will be disseminated to society through the participation of the research and work teams in scientific dissemination activities.
Are any restrictions on data sharing required?	The data may only be used for scientific purposes or for the dissemination of science.

Responsibilities and resources	
Who will be responsible for data management?	Researchers responsible:<br>• Castillo-Navarrete, Juan-Luis; Email: jucastillo@udec.cl<br>• Guzmán-Castillo, Alejandra; Email: aleguzman@ucsc.cl
What resources will you require to develop your plan?	• The IT services and the Directorate of Libraries of the Universidad de Concepción currently have all the necessary resources to meet the requirements of this research data management plan.<br>• The Facultad de Medicina and the Neuroscience, Psychiatry and Mental Health Programme, NEPSAM (http://nepsam.udec.cl) of the Universidad de Concepción have the necessary infrastructure for the development of this plan.

(*) Adapted from:

- 2.3 Componentes de un PGD - Gestión de datos de investigación - Biblioguias at Biblioteca CEPAL, Naciones Unidas [Internet]. Available from: https://biblioguias.cepal.org/gestion-de-datos-de-investigacion/ComponentesPGD
- UdeC. Plan de Gestión de Datos [Internet]. Plan de Gestión de Datos. Dirección de Bibliotecas. 2022. Available from: https://bibliotecas.udec.cl/wp-content/uploads/2022/08/Plan_Gestion_Datos-UdeC.pdf
- DCC. Checklist for a Data Management Plan, v4.0 [Internet]. 2013. Available from: https://dmponline.dcc.ac.uk/files/DMP_Checklist_2013.pdf
